# Prenatal alcohol exposure impairs autophagy in neonatal brain cortical microvessels

**DOI:** 10.1038/cddis.2017.29

**Published:** 2017-02-09

**Authors:** Virginie Girault, Vianney Gilard, Florent Marguet, Céline Lesueur, Michelle Hauchecorne, Yasmina Ramdani, Annie Laquerrière, Stéphane Marret, Sylvie Jégou, Bruno Jose Gonzalez, Carole Brasse-Lagnel, Soumeya Bekri

**Affiliations:** 1 Normandie University, UNIROUEN, INSERM U1245, NeoVasc Team, Rouen, France; 2 Department of Neurosurgery, Rouen University Hospital, Rouen, France; 3 Pathology Laboratory, Rouen University Hospital, Rouen, France; 4 Department of Metabolic Biochemistry, Rouen University Hospital, Rouen, France; 5 Department of Neonatal Pediatrics and Intensive Care and Neuropediatrics, Rouen University Hospital, Rouen, France

## Abstract

Brain developmental lesions are a devastating consequence of prenatal alcohol exposure (PAE). We recently showed that PAE affects cortical vascular development with major effects on angiogenesis and endothelial cell survival. The underlying molecular mechanisms of these effects remain poorly understood. This study aimed at characterizing the ethanol exposure impact on the autophagic process in brain microvessels in human fetuses with fetal alcohol syndrome (FAS) and in a PAE mouse model. Our results indicate that PAE induces an increase of autophagic vacuole number in human fetal and neonatal mouse brain cortical microvessels. Subsequently, *ex vivo* studies using green fluorescent protein (GFP)-LC3 mouse microvessel preparations revealed that ethanol treatment alters autophagy in endothelial cells. Primary cultures of mouse brain microvascular endothelial cells were used to characterize the underlying molecular mechanisms. LC3 and p62 protein levels were significantly increased in endothelial cells treated with 50 mM ethanol. The increase of autophagic vacuole number may be due to excessive autophagosome formation associated with the partial inhibition of the mammalian target of rapamycin pathway upon ethanol exposure. In addition, the progression from autophagosomes to autolysosomes, which was monitored using autophagic flux inhibitors and mRFP–EGFP vector, showed a decrease in the autolysosome number. Besides, a decrease in the Rab7 protein level was observed that may underlie the impairment of autophagosome–lysosome fusion. In addition, our results showed that ethanol-induced cell death is likely to be mediated by decreased mitochondrial integrity and release of apoptosis-inducing factor. Interestingly, incubation of cultured cells with rapamycin prevented ethanol effects on autophagic flux, ethanol-induced cell death and vascular plasticity. Taken together, these results are consistent with autophagy dysregulation in cortical microvessels upon ethanol exposure, which could contribute to the defects in angiogenesis observed in patients with FAS. Moreover, our results suggest that rapamycin represents a potential therapeutic strategy to reduce PAE-related brain developmental disorders.

Prenatal alcohol exposure (PAE) causes various fetal developmental abnormalities, with the brain being particularly affected.^1^ Fetal alcohol syndrome (FAS) combines severe symptoms including intrauterine growth retardation, a characteristic craniofacial dysmorphism, central nervous system developmental abnormalities and behavioral/neurocognitive disturbances. In industrialized countries, FAS is the main cause of acquired, nongenetic mental retardation.^2^ Of note, partial FAS (pFAS) patients are usually not recognized until they present with psychosocial and often neurodevelopmental disabilities during childhood. The most devastating consequence of PAE in the developing brain is neuron loss,^3, 4^ which has an age-dependent window of vulnerability.^5, 6^ Neuronal loss is linked to massive apoptosis mediated by the intrinsic apoptotic pathway via caspase-3 activation.^7^ A recent study suggested that the immature brain is particularly vulnerable to ethanol because of a high expression of proapoptotic proteins and low expression of genes involved in stress response systems such as unfolded protein response or autophagy.^8^ It has also been shown that autophagy activation is a neuroprotective response that alleviates ethanol toxicity.^9, 10^

Autophagy is a process used by cells to recycle long-lived proteins and defective organelles. During this housekeeping process, the intracytoplasmic material is enclosed in double membranes to form autophagosomes that are subsequently delivered to lysosomes for degradation. The autophagic process is highly regulated; several autophagy-related genes (*Atg*) are involved in the formation and maturation of autophagosomes. Microtubule-associated protein light chain 3 (MAP-LC3 or LC3), a mammalian homolog of yeast Atg8, is a component of the autophagosome membrane. During autophagy, the cytosolic LC3-I cytosolic form is lipidized with phosphatidylethanolamine to form LC3-II, which binds to autophagosomal membranes until its degradation in the autolysosome.^11^ LC3-II is therefore used as a marker of autophagic vacuoles. The digestive function of autophagy contributes to cellular homeostasis through quality control for cytoplasmic components. Recent studies have implicated autophagy in cell death by demonstrating many connections to apoptosis, a process with which autophagy shares regulators.^12, 13^ Autophagy is also an adaptive response to nutrient stress that allows for macromolecule synthesis and ATP production.^14^ Interest in autophagy is growing and impairment of this process has been associated with an ever-lengthening list of diseases.^15^ In the brain, defects in the key genes involved in autophagy lead to neurodevelopmental abnormalities.^16^

We recently showed that PAE affects cortical angiogenesis in both mice and FAS patients suggesting that vascular defects contribute to alcohol-induced brain abnormalities.^17^ Importantly, microvessels have recently been shown to have a key role in nerve cell migration.^18^ PAE in mice leads to a lower cortical vascular density, a loss of the microvessel radial orientation and changes in VEGF receptor expression. In humans, stage-dependent changes in the vascular network have been observed in the cortex of fetuses with FAS. No modification was noted between 20 and 22 weeks of gestation (WG), but the radial organization of cortical microvessels was clearly altered in FAS patients from 30 WG. Moreover, it has been demonstrated that alcohol affects angiogenesis in adult nonhuman primates^19^ and mice.^20, 21^ For example, alcohol modifies wound healing and vessel generation by decreasing angiogenesis.^20^ However, the underlying mechanisms are not yet fully understood, and the effects of ethanol on autophagic process have not been yet investigated. This study aimed to investigate the potential effects of PAE on the autophagic process in cortical microvessels from pFAS/FAS human fetuses and from mice prenatally exposed to ethanol. The underlying molecular mechanisms were also investigated in mouse primary microvascular endothelial cells (MPMVEC).

## Results

### PAE induces an increase of autophagic vacuoles number in human fetal and neonatal mouse cortical microvessels

**Figure 1:**
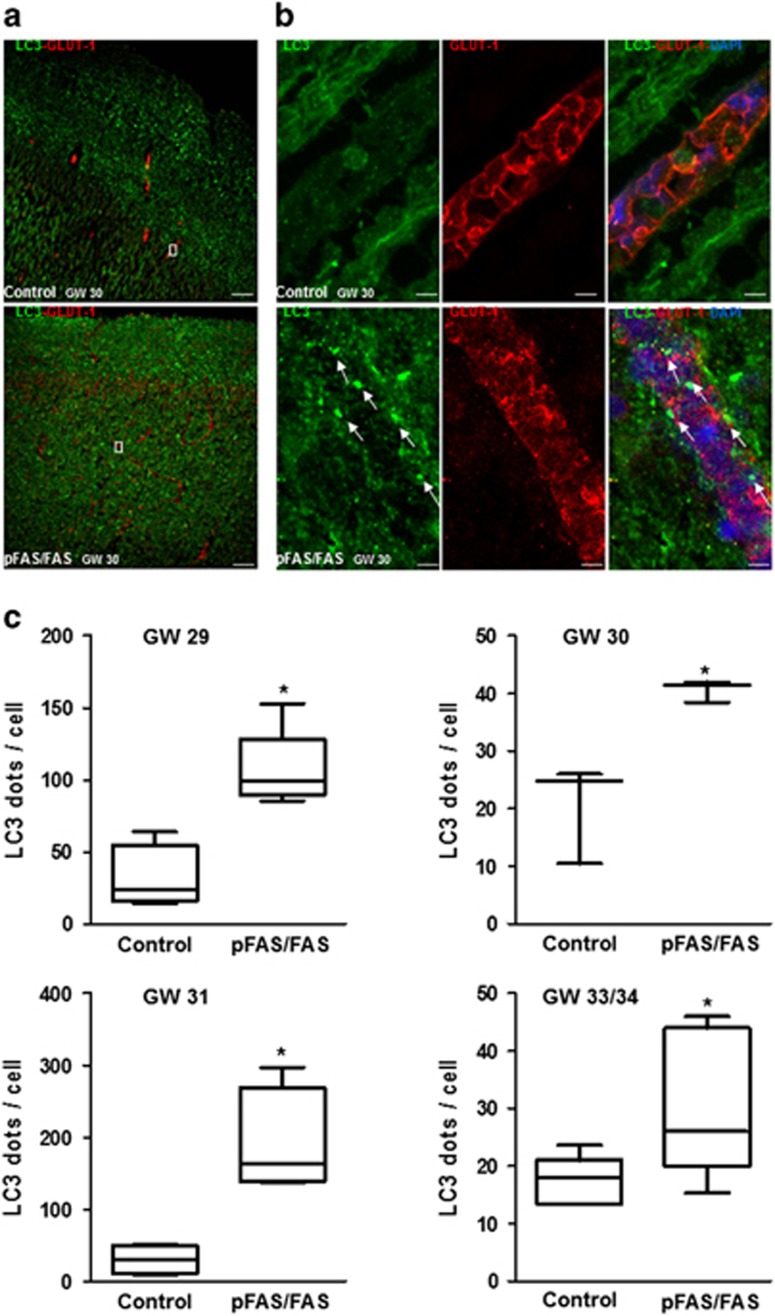
Autophagic vacuole content in brain microvessels of FAS human fetuses observed using confocal microscopy. (**a**) Example from a FAS human fetus at 30 GW with a strong LC3-positive and diffuse green staining in the superficial part of the cortex and disorganization of microvessels (red staining with GLUT1 antibody) compared to a cortical section from a control brain at the same gestational age. The scale bar represents 50 *μ*m. (**b**) At higher magnification: numerous LC3-positive dots in a cortical microvessel of FAS human fetus compared to the control fetus that displayed a less intense and more diffuse LC3 staining. The scale bar represents 5 *μ*m. Arrows indicates LC3-positive dots in endothelial cells. (**c**) Box whisker plot of LC3-positive dots per endothelial cell on confocal images for four FAS and age-matched control fetuses from 29 to 34 WG. Plot depicts the median, interquartile range and range of values of LC3 dots per cell measured in each sample. *P<0.05 *versus* age-matched control, Mann–Whitney test

Four human fetuses at developmental stages ranging from 29 to 34 WG were analyzed by immunohistochemistry using anti-LC3 and anti-GLUT1 (endothelial cell marker) antibodies. This immunolabeling revealed strong LC3-positive staining in the cortex of pFAS/FAS fetuses compared to control cortices (Figure 1a).

Numerous LC3 puncta were observed in cortical microvessels of pFAS/FAS fetuses, whereas cortical microvessels from control fetuses displayed less intense and more diffuse LC3 staining (Figure 1b). The number of LC3-positive dots per endothelial cell was quantified. All pFAS/FAS fetal cortices displayed a significant increase in LC3-positive dots compared to control cases (Figure 1c), suggesting that the number of autophagy vacuoles increases in cortical microvessels upon PAE.

**Figure 2:**
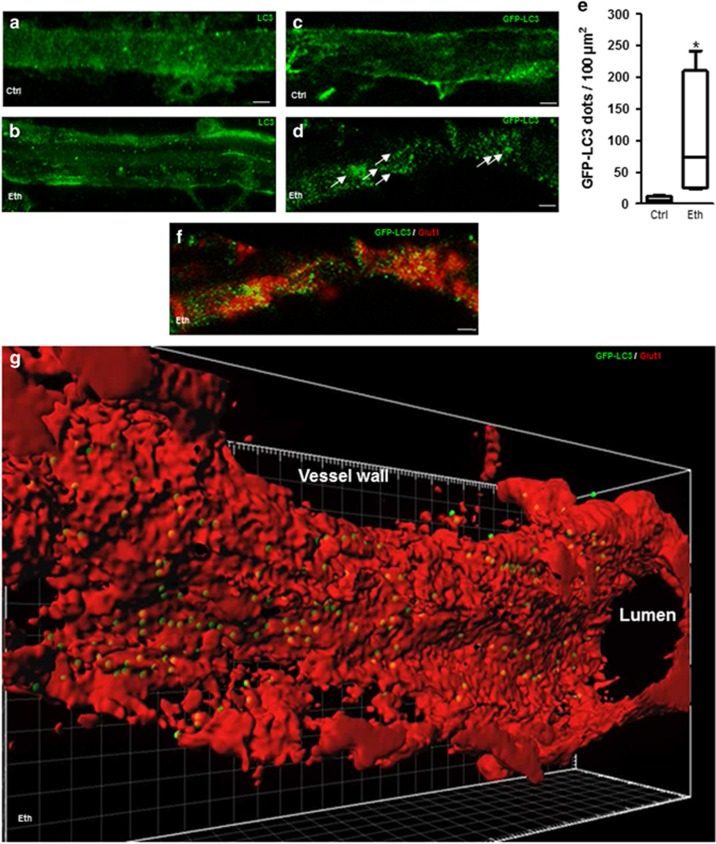
Autophagic vacuole content in mouse brain microvessels observed using confocal microscopy. (**a** and **b**) Daily s.c. injections of sodium chloride (0.9% NaCl) (**a**) or ethanol (3 g/kg, diluted 50% v/v in 0.9% NaCl) (**b**) were administered to pregnant NMRI mice from gestational day 13 to 1 day after parturition. Brain microvessels were extracted from P2 pups and immunolabeled with anti-LC3 antibody. (**c** and **d**) Brain cortical microvessels were extracted from P2 transgenic GFP-LC3 mice and incubated (**c**) without or (**d**) with 50 mM ethanol for 6 h. (**e**) Box whisker plot of GFP-LC3-dots per 100 *μ*m^2^. Plot depicts the median, interquartile range and range of values of LC3 dots measured in each sample. *P<0.05 *versus* age-matched control, Mann–Whitney test. (**f**) Brain microvessels extracted P2 transgenic GFP-LC3 mice incubated for 6 h with 50 mM ethanol were immunolabeled with the anti-GLUT-1 antibody (red). The scale bar represents 10 *μ*m. Arrows indicates LC3-positive dots. (**g**) Data were uploaded into Imaris for three-dimensional (3D) reconstitution and analysis with the Imaris MeasurementPro module

Further experiments were conducted using previously validated model;^17^ pregnant mice received daily s.c. injections of sodium chloride or ethanol. Microvessels were extracted from pup brains at P2 and immunolabeled with anti-LC3 antibody. Confocal microscopy showed that LC3 signal was uniformly distributed in microvessels from control mice while it was restricted to dots in microvessels from animals with PAE (Figures 2a and b).

Besides, cortical microvessels were extracted from the neonatal cerebral cortex of green fluorescent protein (GFP)-LC3 C57BL/6 P2 mice and incubated with 50 mM ethanol for 6 h. GFP-LC3 was uniformly distributed in control conditions while this signal was limited to dots after ethanol exposure (Figures 2c and d). The number of LC3-positive dots was quantified and a significant increase in LC3-positive dots was observed in cells treated with ethanol compared to control cells (Figure 2e). Anti-GLUT-1 antibody staining (Figure 2f) enabled localizing GFP-LC3 puncta within endothelial cells. These data were then uploaded into Imaris for three-dimensional (3D) reconstitution and analyzed with the Imaris MeasurementPro module. This 3D reconstitution indicates that autophagic vacuoles were abundant and located within the microvessel endothelial cells (Figure 2g). This finding is also illustrated by the animation provided in Supplementary Material.

These data suggest that ethanol exposure impairs autophagy in brain cortical microvascular endothelial cells. Therefore, cultured MPMVEC were used to further investigate if the LC3 puncta increase was related to excessive autophagosome production (the generated vacuole number exceeds that of the degraded vacuoles) or the inhibition of autophagic process final steps.^22^

### Effect of ethanol on autophagy activity in brain MPMVEC

**Figure 3:**
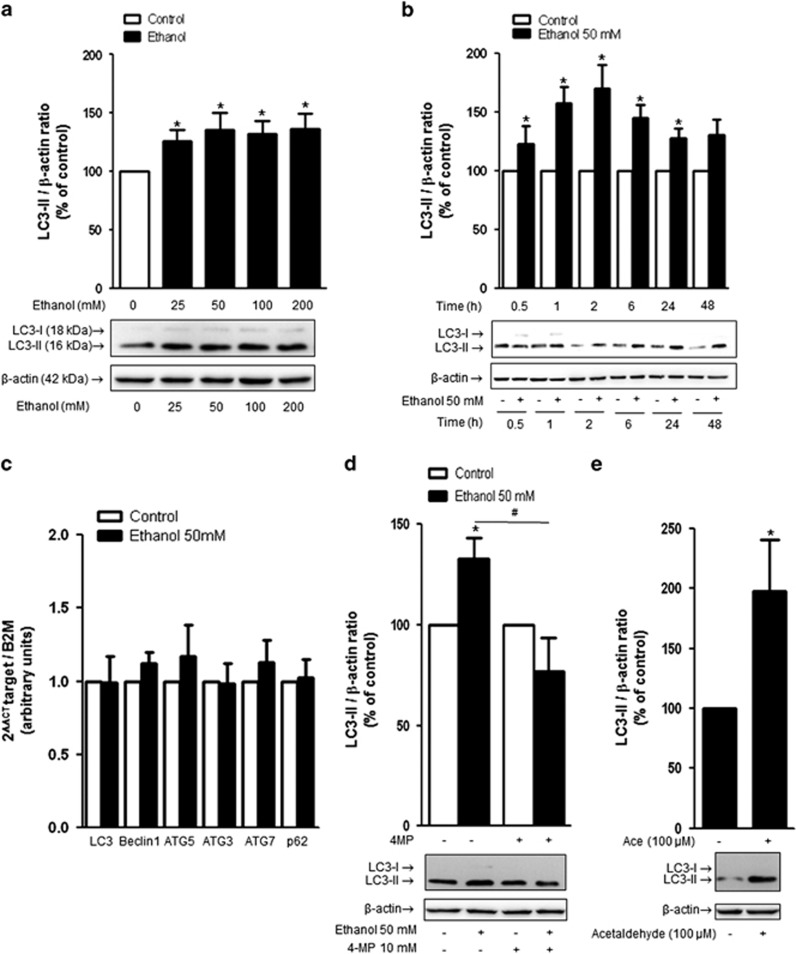
Effect of ethanol on autophagy activity in MPMVEC. (**a**) Influence of various concentrations of ethanol on LC3-II protein level. Western blot of cells incubated for 6 h in the presence or absence of 25, 50, 100 or 200 mM ethanol, showing LC3-I (18 kDa), LC3-II (16 kDa) and *β*-actin (42 kDa). The values are the means±S.E.M. for six independent experiments, expressed as the LC3-II/*β*-actin ratio normalized to 100 for untreated cells. *P<0.05 *versus* control, *n*=6, Student's *t*-test. (**b**) Time course of the effect of 50 mM ethanol on LC3-II protein levels. The values obtained for the quantification of western blots are expressed as means±S.E.M. for the LC3-II/*β*-actin ratio normalized to 100 for untreated cells. *P<0.05 *versus* control at the indicated time, *n*=4, Student's *t*-test. (**c**) Cells were incubated for 2 h in the presence or the absence of 50 mM ethanol, and RT-PCR analysis were performed to determine *LC3*, *Beclin1*, *ATG5*, *ATG3*, *ATG7* and *p62* mRNA levels. Results are expressed as the mean±S.E.M. *P<0.05 *versus* the control set to 1, *n*=5, Student's *t*-test. (**d**) Western blot of LC3 protein from cells cultured for 1 h with 10 mM 4-MP and then with or without 50 mM ethanol for 2 h. The shown values are means±S.E.M. for the LC3-II/*β*-actin ratio normalized to 100 for untreated cells. *P<0.05 *versus* control, ^#^*P*<0.05 *versus* ethanol, *n*=7, Student's *t*-test. (**e**) Western blot of LC3 from cells cultured with or without 100 *μ*M acetaldehyde for 2 h. The shown values are means±S.E.M. for the LC3-II/*β*-actin ratio normalized to 100 for untreated cells. *P<0.05 *versus* control, *n*=6, Student's *t*-test

LC3 immunoblotting using cultured primary MPMVEC derived from neonatal mouse brain allowed us to show that the addition of ethanol at concentrations from 25 to 200 mM to the cultured cells led to an increase in the amount of LC3-II (the autophagosome-associated form; 16 kDa) consistent with a significant increase of autophagic vacuoles (Figure 3a). The effect of 50 mM ethanol was detected at 30 min and peaked at 2 h of ethanol incubation (Figure 3b). The conditions used to further study the effects of ethanol on MPMVEC were 50 mM ethanol incubation for 2 h. Importantly, no significant change in *LC3*, *Beclin1*, *ATG5*, *ATG3* and *ATG7* mRNA levels was observed after 2 h of ethanol exposure (Figure 3c), suggesting that ethanol acts on the autophagic process at a post-transcriptional level.

### Acetaldehyde, the major ethanol metabolite, promotes autophagy disturbances in brain MPMVEC

We investigated whether ethanol or its metabolite, the acetaldehyde, underlies the biological effect. The addition of 4-methylpyrazole (4-MP at 10 mM), which inhibits ethanol oxidation and blocks acetaldehyde formation, prior to the ethanol incubation (2 h with 50 mM ethanol), prevents the ethanol-induced increase of the LC3-II protein level (Figure 3d). In addition, incubation with 100 *μ*M acetaldehyde for 2 h induced a twofold significant increase in the LC3-II protein level (Figure 3e). These data indicate that ethanol, through its transformation into acetaldehyde, induces an increase in the LC3-II protein level at a post-transcriptional stage.

### Influence of ethanol on the initial stages of the autophagic process in brain MPMVEC

**Figure 4:**
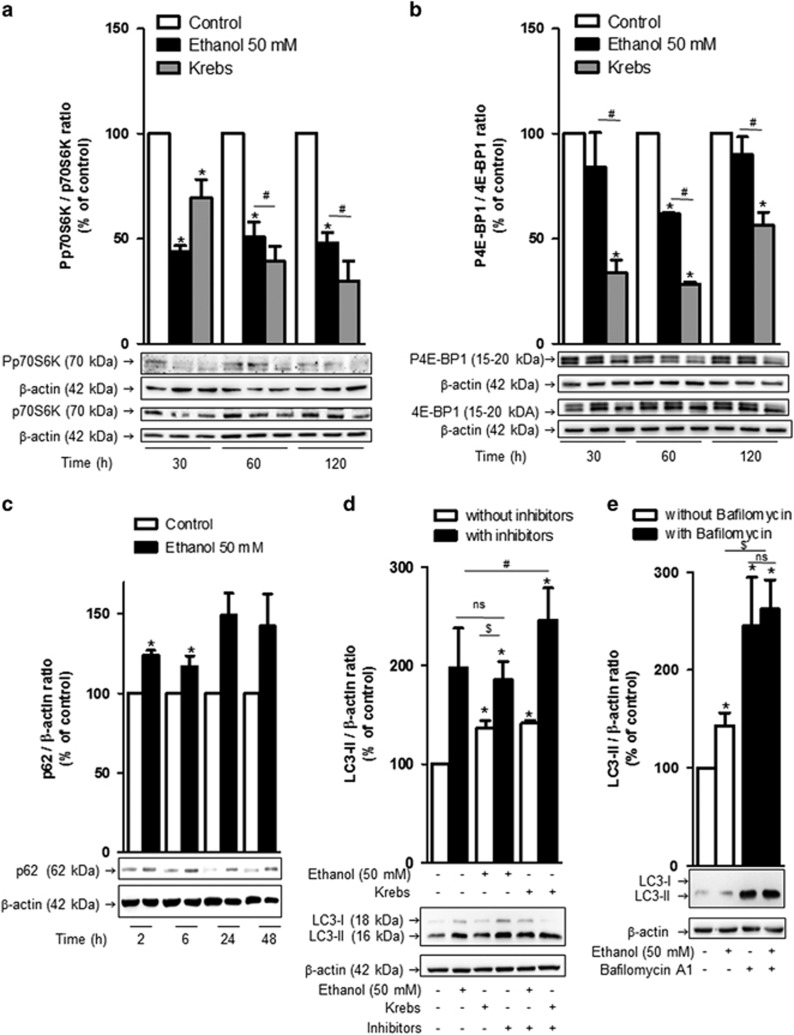
Ethanol impact on autophagic flux in brain microvascular endothelial cells. (**a** and **b**) Western blot analysis of p70S6K and phosphorylated p70S6K (Pp70S6K) or 4E-BP1 and phosphorylated 4E-BP1 (P4E-BP1) protein expression. Cells were incubated upon 0.5, 1 or 2 h with ethanol or Krebs medium, and western blot analyses were performed. Values are means±S.E.M. for three independent experiments and expressed as Pp70S6K/p70S6K or P4EBP-1/4EBP-1 ratio normalized to 100 for untreated cells. *P<0.05 compared with control, Student's *t*-test. ^#^*P*<0.05 compared with Krebs medium at the same time analyzed, Student's *t*-test. (**c**) Western blot analysis was performed to determine p62 protein levels after 2, 6, 24 or 48 h of ethanol exposure; the values shown are means±S.E.M. for four independent experiments and are expressed as the p62/*β*-actin ratio normalized to 100 for untreated cells, for the indicated times. *P<0.05 *versus* control; *n*=4, Student's *t*-test. (**d** and **e**) Analysis of the autophagic flux. Cells were incubated for 1 h with protease inhibitors (**d**) or Bafilomycin A1 (**e**) and then with or without 50 mM ethanol or Krebs medium for 2 h. Western blot analysis were performed to determine LC3-II protein level. The values shown are means±S.E.M. for four independent experiments and are expressed as the LC3-II/*β*-actin ratio normalized to 100 for untreated cells. *P<0.05 *versus* control; ^$^*P*<0.05 *versus* ethanol alone; ^#^*P*<0.05 *versus* the control with protease inhibitors; NS, nonsignificant; *n*=4 or *n*=5 with ethanol, Student's *t*-test

The LC3-II level increases as a result of enhanced formation or decreased autophagosome turnover. Inhibition of the mammalian target of rapamycin (mTOR) signaling pathway, a central regulator of cell metabolism, growth, proliferation and survival, activates autophagy by increasing autophagosome formation. To determine the effect of ethanol on this pathway, western blot analyses focusing on 70 kDa ribosomal S6 kinase (p70S6K) and translation repressor protein 4E-BP1, which are phosphorylated by mTOR, were performed. As shown in Figures 4a and b, a significant decrease in phosphorylated p70S6K was observed after 30 min of incubation with both ethanol and Krebs medium, a starvation condition known to activate autophagy by inhibiting the mTOR pathway and decreasing p70S6K phosphorylation. However, after 1 or 2 h of incubation, ethanol had a lower effect than that observed with Krebs medium. Phosphorylation of 4E-BP1 decreased significantly after 1 h. These results indicate that mTOR inhibition could be involved, at least in part, in the ethanol-induced increase in autophagic vacuoles number.

### Ethanol effect on autophagic flux in brain MPMVEC

The ethanol effect on autophagy activity was investigated by studying the level of sequestosome 1. Sequestosome 1 (p62) is a scaffolding protein that interacts with LC3 and is degraded following the fusion between autophagosomes and lysosomes.^23^ Immunoblot experiments revealed a progressive and significant increase in the level of p62 protein in cells cultured with 50 mM ethanol (Figure 4c), without the p62 mRNA level being affected (Figure 3c). Thus, ethanol inhibits p62 protein degradation within the autolysosomes, suggesting that ethanol blocks or reduces the autophagic vacuole turnover.

To confirm these results, autophagic flux assays were performed by inhibiting the degradation of autolysosome content. Thus, LC3 turnover was assessed in the presence of lysosomal protease inhibitors, E64d and pepstatin A. Cells were treated with these inhibitors for 1 h, and then with 50 mM ethanol or Krebs medium for 2 h. As expected, a higher level of LC3-II protein was observed in cells cultured with protease inhibitors or with Krebs medium compared to cells cultured with the control medium. Basal autophagy is significantly higher in ethanol-treated cells compared to control cells (Figure 4d). There is a significant difference of LC3-II level between ethanol condition and ethanol plus protease inhibitors. However, no further significant increase in the protein level was observed in cells cultured with ethanol and protease inhibitors compared to control cells treated with inhibitors alone. Besides, the induction of autophagic vacuole formation upon starvation conditions was confirmed by a significant increase in the LC3-II protein level in cells cultured with Krebs medium along with protease inhibitors compared to cells treated with inhibitors alone (Figure 4d). Treatment with Bafilomycin A1, a strong inhibitor of the vacuolar type H^+^-ATPase that blocks the fusion step of the autophagic process allows to yield similar results with no difference between cells treated with Bafilomycin and cells treated with ethanol and Bafilomycin (Figure 4e).

**Figure 5:**
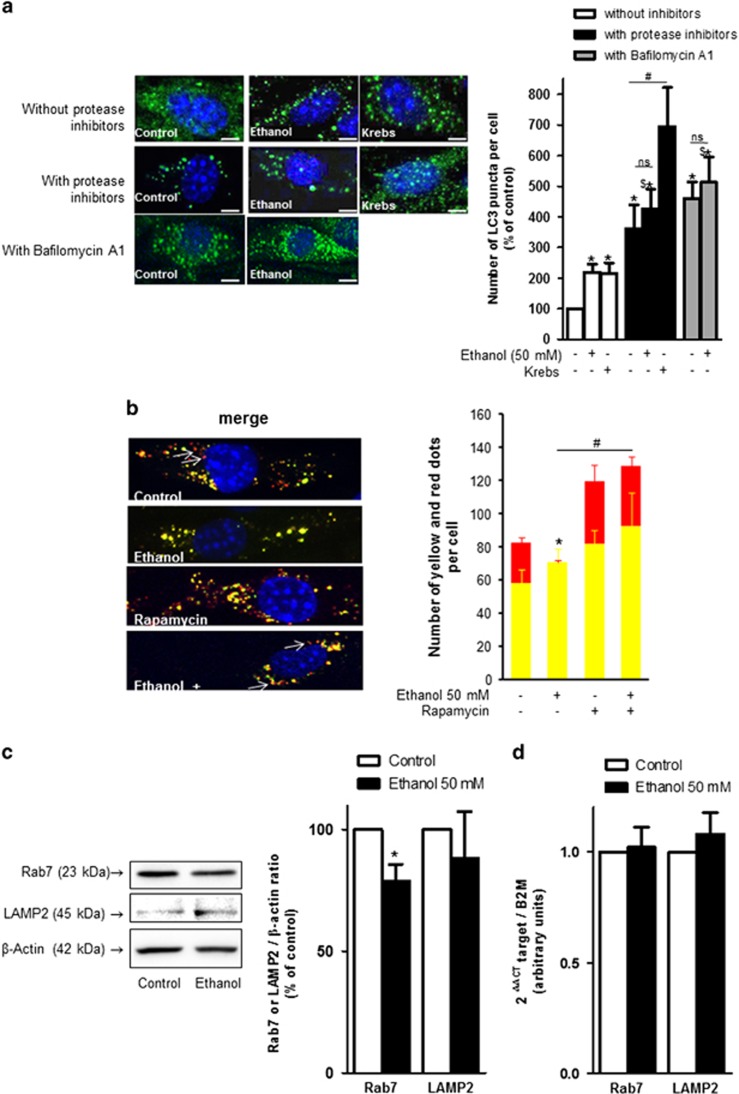
(**a**) Confocal microscopy of mouse primary brain microvascular endothelial cells cultured with or without protease inhibitors or Bafilomycin A1, and then with or without ethanol (50 mM) or Krebs medium for 2 h. Immunolabeling was then performed with the LC3 antibody (green) and nuclei were stained with Hoechst (blue). The scale bar represents 5 *μ*m. The number of green dots (LC3 staining) per cell is normalized to 100 for untreated cells and is presented as the mean±S.E.M. *P<0.05 *versus* control, ^#^*P*<0.05 *versus* control with protease inhibitors, ^$^*P*<0.05 *versus* ethanol alone, NS, nonsignificant, *n*=4, Student's *t*-test. (**b**) Confocal microscopy of mouse primary brain microvascular endothelial cells transfected with the TagRFP-EmGFP-LC3 vector; 48 h after transfection, cells were cultured with or without ethanol (50 mM) for 2 h, and then overnight with protease inhibitors with or without rapamycin 200 nM. Red dots indicate autolysosomes, whereas yellow dots indicate autophagosomes. The scale bar represents 10 *μ*m. The number of green dots (LC3 staining) per cell is presented as the means±S.E.M. for three independent experiments and are expressed as the number of red and yellow dots per cell. *P<0.05 *versus* control, *n*=3, Student's *t*-test. (**c**) Mouse primary brain microvascular endothelial cells were incubated for 2 h with or without ethanol (50 mM), and western blot analysis was performed to determine the Rab7 and LAMP2 protein levels. Representative autoradiograms are presented and the values are the means±S.E.M. for five independent experiments, and are expressed as the Rab7 or LAMP2/*β*-actin ratio normalized to 100 for untreated cells. *P<0.05 *versus* control, *n*=5, Student's *t*-test. (**d**) RT-PCR analyses were performed to determine *Rab7* and *LAMP2* mRNA levels after 2 h of ethanol incubation. Results are expressed as the mean±S.E.M. *P<0.05 *versus* the control, set to 1, *n*=4, Student's *t*-test

LC3 immunofluorescence assay revealed that the LC3-positive puncta density was two fold higher in cells incubated with 50 mM ethanol for 2 h compared to control cells (Figure 5a). In these experiments, a significant increase was also observed in cells cultured with ethanol and protease inhibitor or Bafilomycin A1 compared to cells treated with ethanol alone. However, the number of LC3-positive dots was not significantly higher in cells cultured with ethanol and protease inhibitors or Bafilomycin A1 compared to cells cultured with protease inhibitors alone or Bafilomycin A1. These data suggest that ethanol induces a partial block in autophagic flux.

### Ethanol impairs autophagosome–lysosome fusion in brain MPMVEC

Tandem fluorescence-tagged LC3 reporters (TagRFP-EmGFP-LC3) is used for monitoring autophagic flux in living cells to enable differentiating autophagosomes from autolysosomes.^24^ The acidic environment within the lysosome quenches EmGFP green fluorescent signal, but has no effect on that of TagRFP. On merged images, yellow dots (i.e., RFP^+^GFP^+^) correspond to autophagosomes, whereas red dots (i.e., RFP^+^GFP^−^) indicate autolysosomes. In these experiments, MPMVEC were incubated for 2 h with or without 50 mM ethanol, then overnight with protease inhibitors to prevent the degradation of autophagic vacuoles allowing, thus, their visualization. The number of red dots was significantly lower in the presence of ethanol (Figure 5a), with a significant decrease of the red/green dot ratio compared to control cells (Figure 5b). The smaller number of autolysosomes (red dots) in ethanol-treated cells compared to control cells suggests that ethanol exposure impairs autophagic flux by decreasing the rate of fusion between autophagosomes and lysosomes. Interestingly, when rapamycin, an mTOR inhibitor that activates the autophagic process, was added to endothelial cell cultures after 2 h of ethanol incubation, some red dots were observed anew indicating that rapamycin can restore autophagic flux and promote the formation of autolysosomes that was impaired by ethanol. Of note, the number of red dots in the presence of rapamycin alone was similar to that of control cells (Figure 5b).

We then studied the expression of the lysosomal proteins Rab7 and LAMP2 involved in the autolysosome formation. Western blotting showed that in cells exposed to ethanol, the level of Rab7 was mildly but significantly reduced compared to that of cells cultured in control conditions. No differences in the LAMP2 protein level (Figure 5c) or the *Rab7* or *LAMP2* mRNA levels (Figure 5d) were observed. Thus, in endothelial cells, Rab7 could be specifically targeted upon ethanol-induced inhibition.

### Modulation of autophagy prevents cortical microvessel damages and MPMVEC death induced by ethanol exposure

**Figure 6:**
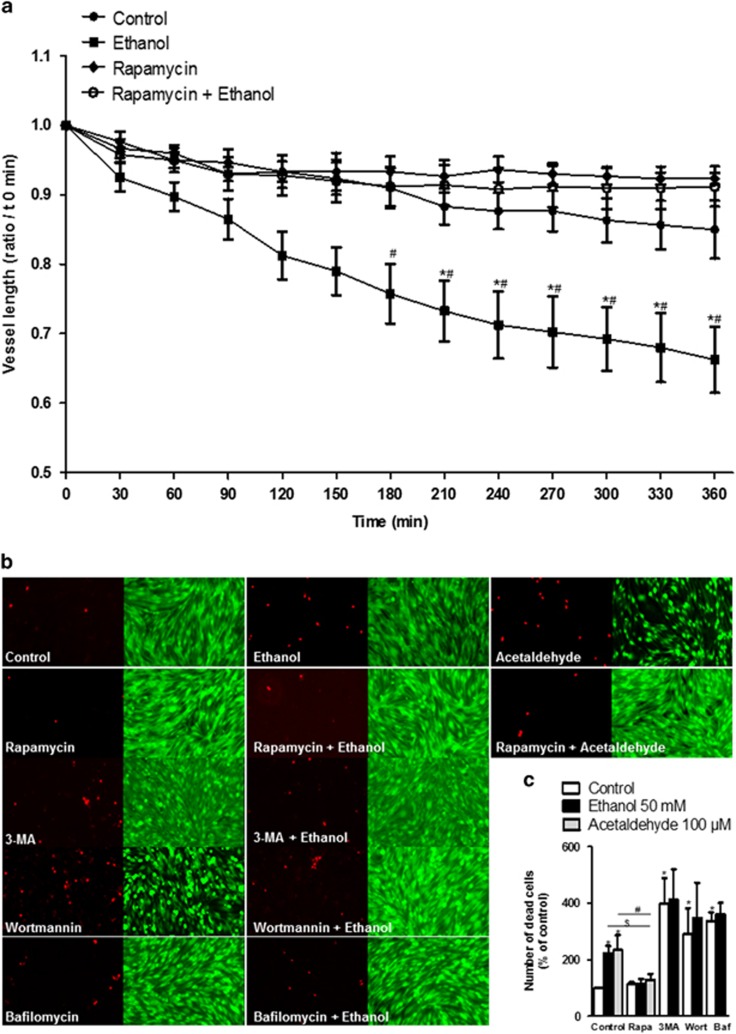
Effect of ethanol on endothelial cell death and brain cortical microvessel plasticity. (**a**) Effect of ethanol on microvessel plasticity in cultured brain slices from P2 animals. Time course of the effect of 50 mM ethanol on brain microvessel length in the presence or absence of 200 nM rapamycin. *P<0.05 *versus* control at the indicated time; ^#^*P*<0.05 *versus* rapamycin+ethanol at the indicated time; two-way ANOVA; *n*=4. (**b**) The endothelial cells were labeled with calcein AM (green) and ethidium D1 (red) after 6 h of exposure to 50 mM ethanol or acetaldehyde 100 *μ*M, in the presence or absence of 200 nM rapamycin, 30 mM 3-methyladenin (3-MA), 75 nM wortmannin (Wort) or 100 nM Bafilomycin A1 (Baf). Scale bar: 40 *μ*m. (**c**) Quantification of ethidium labeling cells with ImageJ software. *P<0.05 *versus* control; ^$^*P*<0.05 *versus* ethanol 50 mM; ^#^*P*<0.05 *versus* acetaldehyde 100 *μ*M, Student's *t*-test, *n*=4

Since ethanol has previously been shown to affect vascular plasticity and to promote microvessel cell death,^17^ we investigated the effect of different modulators of autophagy on ethanol-related damages. As expected, time-lapse acquisition showed that ethanol exposure reduces the vascular plasticity and thus vessel length, while the treatment with rapamycin prevented this effect (Figure 6a). Of note, no effect was observed with rapamycin alone. In addition, rapamycin decreased the rate of cell death induced by either ethanol or acetaldehyde (Figures 6b and c). Bafilomycin A1 and two inhibitors of autophagy initiation steps (3-methyladenine and wortmannin) induced a very significant increase in the number of dead cells. No further increase upon ethanol exposure was observed in the presence of these inhibitors (Figures 6b and c).

Taken together, these results showed that endothelial cells are very sensitive to autophagosome accumulation and that the ethanol toxicity may be prevented by rapamycin.

### Ethanol enhances the nuclear translocation of apoptosis-inducing factor

**Figure 7:**
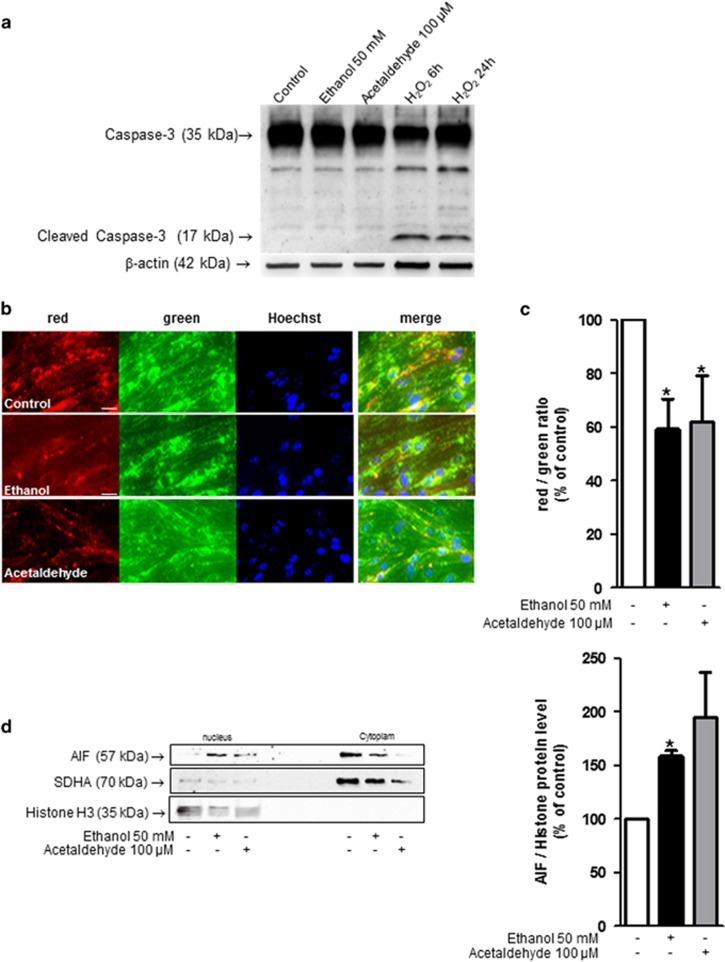
Effect of ethanol on caspase-independent apoptosis in endothelial cells. (**a**) Western blots analysis of cleaved caspase-3 after 6 h of exposure to 50 mM ethanol, *n*=3. H_2_O_2_ (1 mM) was used as a positive control after 6 and 24 h of exposure. (**b**) Integrity of mitochondrial membrane potential was investigated by a mitochondrial potential-sensitive dye, JC-1. When this dye enters mitochondria, the color changes from red to green. Hoechst was used to label nuclei (blue). Scale bar: 20 *μ*m. (**c**) The values are the means±S.E.M. for three independent experiments and are expressed as red/green ratio. *P<0.05 *versus* control, Student's *t*-test. (**d**) Western blot analysis of AIF protein expression in the nucleus and cytoplasm. Cells were incubated 2 h with or without 50 mM ethanol or 100 *μ*M acetaldehyde, nuclear and cytoplasm protein were extracted, and western blot performed. Mitochondrial contamination of nuclear extract is checked by western blot analysis using anti-succinate dehydrogenase complex, subunit A (SDHA) antibody. Values are means±S.E.M. for three independent experiments and expressed as AIF/histone ratio normalized to 100 for untreated cells. *P<0.05 compared with control, Student's *t*-test

To clarify the mechanisms of ethanol toxicity, we investigated apoptosis pathways. Ethanol-induced cell death is likely to be caspase-3 independent since the level of cleaved caspase-3 protein was not higher in ethanol-treated cells for 6 h compared to control cells (Figure 7a). JC-1, a cationic dye, selectively enters mitochondria upon a decrease of membrane potential, causing a reversible change of their color from red to green.^25^ A decrease of red puncta dots was observed in cells incubated with either ethanol or acetaldehyde (Figures 7b and c). Apoptosis-inducing factor (AIF) is a caspase-independent death effector that induces chromatin condensation and DNA fragmentation when translocated into the nuclei.^26^ A significant increase in AIF nuclear translocation was observed in cells that were incubated with either ethanol or acetaldehyde (Figure 7d). Ethanol or acetaldehyde conceivably induces endothelial cell death through a caspase-independent pathway mediated by mitochondrial release of AIF and its nuclear translocation.

## Discussion

In this study, we investigated whether the observed ethanol-related damages of microvessel endothelial cells in the developing brain was associated with impaired autophagy.

A significant autophagic vacuole accumulation was observed in pFAS/FAS human and mouse brain cortical microvessels following PAE. This accumulation may favor the formation of intracellular aggregates and likely hastens cell injury and death. The accumulation of autophagic vacuoles may contribute to the vascular network disturbances observed in both humans and mice upon PAE. However, little is currently known about the role of autophagy in vascular biology, particularly in the brain.^27^ In the human brain, arterial and venous development starts at 6 WG, at the same time as neurogenesis and both processes require tight regulation.^28^ From the initial meningeal vascular plexus, which covers the entire surface of the embryonic brain, six groups of vessels perforate the glia limitans and develop radially and perpendicularly to the surface. The length of these vessels increases as the cortex increases in thickness. Blood supply to the white matter is provided by group 6 from 15 WG, whereas group 1 vascularizes the superficial 27 WG onward. The other groups form an intracortical microvascular network between 20 and 27 WG, by means of divisions, branching or coiling. We have previously shown that this network was disrupted in pFAS/FAS fetuses during the third trimester.^17^

Several lines of evidence link autophagy and angiogenesis. Autophagy has been identified as a novel target for enhancing the therapeutic efficacy of angiogenesis inhibitors.^29, 30, 31^ Angiogenesis plays a critical role in the pathology of various diseases including cancer, and a number of strategies are being developed to inhibit tumoral angiogenesis. However, a complex relationship exists between autophagy and angiogenesis. Some studies have suggested that autophagy inhibits angiogenesis,^29, 32^ whereas others have suggested that autophagy promotes it.^30, 33^ Currently, the involvement of disrupted autophagy in the effect of ethanol on angiogenesis remains unknown. In pFAS/FAS children, it could be hypothesized that PAE impairs the autophagic process in endothelial cells and modifies the vascular network, which could in turn contribute to the neurodevelopmental abnormalities by interfering with neuronal migration.

The molecular mechanisms involved in ethanol-induced autophagy vacuole accumulation in brain cortical microvessels were characterized. Ethanol effects are mediated by its metabolite, acetaldehyde. Thus, the inhibition of ethanol oxidation by 4-MP completely prevents the LC3-II increase. Moreover, acetaldehyde addition mimics LC3-II protein accumulation induced by ethanol. Ethanol is predominantly metabolized in the liver, and previous studies demonstrated that acetaldehyde is involved in hepatic injury and impaired autophagy.^34^ Several steps of the autophagic process are targeted in endothelial cells following ethanol exposure. The initial step is altered through a partial inhibition of the mTOR pathway. This protein is a 289 kDa serine–threonine kinase, which forms at least two distinct multi-protein complexes, mTOR complex 1 (mTORC1) and mTOR complex 2 (mTORC2).^35^ mTORC1 is known to regulate autophagy by modulating specific proteins that control the autophagy machinery. The phosphorylations of P70S6K and 4E-BP1 have been used as a hallmark of mTORC1 activation and are correlated with autophagy inhibition in various situations. The ethanol-related inhibition of P70S6K and 4E-BP1 phosphorylation in endothelial cells indicates that ethanol suppresses mTORC1 activity. The ethanol effect on mTOR pathway had previously been demonstrated in neuronal cells and in other cell types such as hepatocytes and cardiomyocytes.^36, 37, 38^ It has been demonstrated in neurons that ethanol toxicity is mediated by reactive oxygen species (ROS) production and that autophagy is activated to reduce the subsequent oxidative stress.^9^ We demonstrated, in this study, that in MPMVEC, ethanol impairs mitochondrial integrity and induces AIF protein release as well as its nuclear translocation, which may induce programed cell death.

The final autophagy steps allowing for autolysosome formation are also impaired, with a decrease in Rab7 level, a protein that is required for the autophagosome–lysosome fusion. The late endosomal compartment proteins Rab7 and LAMP2 have been shown to be involved in the late stages of autophagosome maturation, presumably facilitating the fusion of autophagosomes with late endosomes.^39^ Previous studies have shown that impaired autolysosome formation was correlated with LAMP2 depletion and a relative paucity in autolysosomes.^40, 41^ In hepatocytes, it has recently been shown that ethanol increases the LC3-II level and decreases LAMP2 and Rab7 levels, potentially contributing to ethanol-induced liver injury.^42^ However, in neonatal cortical endothelial cells, we observed no effect of ethanol exposure on LAMP2 level. Thus, in this cell type, the specific decrease in the Rab7 protein level may contribute to the accumulation of autophagic vacuoles, but the mechanism underlying the decrease of this protein, which occurs at a post-transcriptional level, remains to be elucidated. Altogether, the mechanisms involved at different steps of autophagy very likely contribute to the accumulation of autophagic vacuoles in endothelial cells.

We have shown that autophagy activation using rapamycin, an mTOR inhibitor, decreases ethanol-induced endothelial cell death and restores the vascular plasticity. This finding appears to be contradictory to the mTOR partial inhibition observed upon ethanol exposure. However, the effect of ethanol on p70S6K and 4E-BP1 phosphorylation does not last as long as the inhibition observed in the starvation condition. The mTOR subcellular localization has been reported to enable its effects on the spatial and temporal control of cell homeostasis.^43^ Thus, mTOR modulation of autophagy is not fully understood, and its kinetics and the subcellular localization of its targets can have a great impact. One may hypothesize that this partial activation of the initial steps of authophagy upon ethanol treatment may be related to a protective cellular response to ethanol injury. The protective effect of autophagy observed in endothelial cells was previously reported in neurons in which autophagy acted as a protective response against ethanol neurotoxicity.^10^ In SH-SY5Y cells, the autophagy inhibition promotes ROS generation and leads to higher levels of ethanol-induced cell death. In addition, ethanol exposure activates mitophagy, suggesting that the neuroprotection provided by autophagy may result from the removal of damaged mitochondria.^9^ Moreover, Pla *et al.*^44^ showed that autophagy protects against ethanol toxicity in mouse astrocytes and neurons. Thus, autophagic flux activation by rapamycin may be hypothesized to protect both neurons and endothelial cells against ethanol-induced death. More recently, peritoneal injection of rapamycin was proposed to prevent neuroinflammation and neuronal death in a mouse model of cerebral palsy.^45^ Further experiments are needed to characterize the involvement of autophagy in brain angiogenesis and the *in vivo* protective effect of rapamycin after PAE.

To summarize, we have shown for the first time that PAE impairs autophagy in brain endothelial cells, thereby contributing to alterations in angiogenesis and to the subsequent brain abnormalities observed in pFAS/FAS patients. Our results strongly suggest that rapamycin represents a potential therapeutic strategy to reduce brain developmental disorders related to PAE.

## Materials and Methods

### LC3/Glut1 confocal microscopy in human cerebral cortices

Eight fetal brains ranging from 29 to 34 WG were subdivided in two groups. Four brains belonging to the control group were obtained from fetuses whose brain was macroscopically and microscopically free of detectable abnormalities. Four brains from pFAS/FAS fetuses were obtained after spontaneous *in utero* death (Supplementary Tables 1 and 2).

Seven micrometer tissue sections from the frontal cortex were deparaffinized in xylene and rehydrated in decreasing concentrations of ethanol. Antigen retrieval was performed by heating the sections in 10 mM boiling sodium citrate buffer (pH 6.0) for 15 min. Then, sections were incubated overnight at 4 °C with anti-GLUT1; 1/200 (sc-1605, Santa Cruz Biotechnology, Dallas, TX, USA) and anti-LC3; 1/200 (PM036, MBL International Corporation, Woburn, MA, USA) as previously described.^17^ Afterwards, sections were incubated with fluorescent-conjugated antibodies (Alexa Fluor 594-conjugated donkey anti-goat; 1/400 (A11058, Life Technologies, Waltham, MA, USA), Alexa Fluor 488-conjugated donkey anti-rabbit; 1/400 (A21206, Life Technologies)). Confocal images were acquired with the Leica laser scanning confocal microscope TCS SP2 AOBS (Leica Microsystems, Wetzlar, Germany).

### Animals

NMRI (Naval Medical Research Institute) mice were purchased from Janvier (Le Genest Saint Isle, France), and GFP-LC3 transgenic mice, reporter mice systemically expressing GFP fused to LC3, were obtained from the RIKEN BioResource Center (Ibaraki, Japan).^46^ The mice were reared in controlled temperature rooms (21±1 °C) with a 12 h light/12 h dark cycle and free access to food and water. Pups were killed at 2 days of age (P2) and their brain cortical microvessels were extracted. Animal manipulations were performed according to the recommendations of the European Communities Council Directives (86/609/EEC) and the French National legislation (ethical approval no. 01680.02), and were supervised by authorized investigators (BJG, authorization no. 7687 from the French Ministry of Agriculture and Fisheries).

### *In vivo* treatment of pregnant mice and newborns

Pregnant NMRI mice received a daily s.c. injection of sodium chloride (0.9% NaCl) or ethanol (3 g/kg, VWR, 83813.360) diluted (50% v/v) in 0.9% NaCl, as previously described.^17^ Injections were performed from gestational day 13 (GD13) to GD19. Mothers received a single injection of the same treatment at parturition (P0) and 1 day after parturition (P1). The pups were killed on P2.^17^

### Isolation of brain cortical microvessels in mice

The heads of P2 NMRI mice prenatally exposed to ethanol or those of P2 transgenic GFP-LC3 transgenic mice were washed in 70% ethanol, and the cortices were extracted in culture medium (endothelial basal medium supplemented with 2% FBS (CVFSVF00-01, Eurobio, Courtabeuf, France) and 1% antibiotics and antimycotics (P11-002, PAA, Velizy-Villacoublay, France)). The meninges were removed, and the cortices were lysed in 7 ml of culture medium, in a Dounce tissue homogenizer. The lysate was centrifuged (420 × *g*, 15 min, 4 °C), and the pellet was suspended in 18% (w/v) dextran solution. This suspension was centrifuged (30 min, 2000 × *g*, 4 °C), and the pellet was suspended in the culture medium and then filtered through a 70 *μ*m cell strainer (352350, BD Biosciences, San Jose, CA, USA). The resulting suspension of microvessels was centrifuged (7 min, 550 × *g*, 4 °C), the pellet was washed in PBS (BP3994, Fisher Bioreagents, Illkirch, France) and the resulting suspension was centrifuged again (7 min, 550 × *g*, 4 °C). The cortical microvessels extracted from P2 NMRI mice were cytospin on a glass slide and fixed by overnight incubation in 4% PFA (sc-281692, Santa Cruz Biotechnology) for immunohistochemistry. Before the cytospin, the cortical microvessels extracted from P2 transgenic mice were treated for 6 h at 37 °C with artificial cerebrospinal fluid medium (aCSF) containing 125 mM NaCl, 3 mM KCl, 2 mM CaCl_2_, 1.2 mM NaHPO_4_, 26.2 mM NaHCO_3_, and 10 mM d-glucose, pH 7.4. The aCSF was used alone or together with 50 mM ethanol.

### Endothelial cell culture

C57BL/6 mouse primary brain microvascular endothelial cells isolated from brain tissue of P2 pups were purchased from Cell Biologics (Chicago, IL, USA). Cells were cultured at 37 °C, in a humidified environment, in the presence of 5% CO_2_. They were fed every 2 days with a complete mouse endothelial cell culture medium, on 60 mm diameter dishes or 12-well slides. Two days after confluence was reached, the cells were incubated for 24 h with serum-free medium. The cells were then treated with various pharmacological agents, including 50 mM ethanol, 100 *μ*M acetaldehyde, lysosomal protease inhibitors 10 *μ*g/ml pepstatin A (P-5318, Sigma-Aldrich, St Louis, MO, USA) and 10 *μ*g/ml E64D (sc-201280 A, Santa Cruz Biotechnology), 200 nM rapamycin (R0395, Sigma-Aldrich), 100 nM Bafilomycin A1 (B-1793, Sigma-Aldrich), 10 mM 4-methylpyrazole (4-MP, M-1387, Sigma-Aldrich), 30 mM 3-methyladenine (M9281, Sigma-Aldrich), 75 nM wortmannin (W3144, Sigma-Aldrich), Krebs medium (145 mM NaCl, 4.75 mM KCl, 1.18 mM KH_2_PO_4_, 1.18 mM MgSO_4_ hydrate, 25 mM NaHCO_3_, 2.5 mM CaCl_2_, pH 7.4) – this medium is commonly used for starvation condition, and ethanol (25 to 200 mM).

### Transfection with the tandem sensor mRFP–EGFP–LC3 probe

We used the autophagy tandem sensor mRFP–EGFP–LC3 probe (P36269, Life Technologies) to determine the localization of the LC3 protein and thus the autophagic flux in cultured endothelial cells. This probe contains an acid-sensitive GFP and an acid-insensitive RFP. Since the autophagosome has a neutral pH and the autolysosome is acidic, the maturation of autophagosomes into autolysosomes is accompanied by a loss of GFP fluorescence. The endothelial cells were cultured to 80–90% confluence for transfection. Single-cell suspensions were prepared at a density of 1.0 × 10^4^/ml and used to seed 12-well slides containing 1 ml of medium per well. Transfection was performed in accordance with the kit manufacturer's instructions. In brief, 6 *μ*l of lipofectamine LTX (15338-100, Life Technologies) was added together with 2 *μ*l of Plus Reagent and 14 *μ*l of the tandem sensor mRFP–EGFP–LC3 probe, to 200 *μ*l of opti-MEM reduced-serum medium (31985-062, Life Technologies). The resulting transfection mixture was mixed gently and incubated for 5 min at room temperature. The mRFP–EGFP–LC3 probe-Lipofectamine LTX mix was added to the wells containing the cells and opti-MEM. The cells were incubated for 4 h at 37 °C. Subsequently, complete endothelial cell medium was added for a further 48 h incubation. The cells were incubated with serum-free medium for a final 24 h. Cells were then treated for 2 h with 50 mM ethanol and overnight with lysosomal protease inhibitors (10 *μ*g/ml E64D+10 *μ*g/ml pepstatin A).

### Endothelial cell immunolabelings

Endothelial cells or brain cortical microvessels were washed three times with PBS and fixed by incubation in 4% PFA in PBS for 1 h at room temperature. After incubation with 1% BSA/0.3% Triton X-100 in PBS to block nonspecific sites, primary antibodies (anti-LC3; 1/200 (PM036, MBL International Corporation), anti-GLUT1; 1/200 (sc-1605, Santa Cruz Biotechnology)) were added, and the preparations were incubated overnight at 4 °C. Secondary antibodies in blocking solution were then incubated with the preparations for 1 h at room temperature (Alexa Fluor 594-conjugated donkey anti-goat; 1/400 (A11058, Life Technologies), Alexa Fluor 488-conjugated donkey anti-rabbit; 1/400 (A21206 Life Technologies)).

### Laser scanning confocal microscopy and reconstitution 3D images

The confocal images were collected with a TCS SP2 AOBS laser scanning confocal imaging system (Leica Microsystems) equipped with × 40 and × 63 high numerical-aperture oil immersion objective lenses. Image size was set to 1024 × 1024 pixels. Multitrack sequential acquisition settings were used to prevent interference between channels. A 561 nm diode solid-state laser and the 488 nm line of an argon ion laser were used for excitation. Emission detection bandwidths were optimized with Leica software. The confocal pinhole was set to 1 Airy unit. Z-stack acquisition intervals were selected so as to satisfy Nyquist sampling criteria. As described in the Imaris version 7.6 reference manual (Bitplane, South Windsor, CT, USA), Imaris software provides eight different viewing functions for the generation and visualization of high-quality confocal images. In this project, confocal Z-stacks comprising up to 40 images were reconstructed into 3D animations with the aid of Imaris software in the Slice and Easy 3D modes. The Spot function of Imaris automatically locates autophagosomes on the basis of size and intensity thresholds. Each autophagosome is represented by a sphere of arbitrary size determined by the user. The sphere is displayed at the center of mass of the identified autophagosome.

### Subfractionization of cellular proteins

Total protein content was extracted using the endothelial cells. After treatment, the cells were washed three times on ice with PBS and their protein content was extracted in lysis buffer (50 mM Hepes, 150 mM NaCl, 10 mM EDTA, 10 mM glycerophosphate, 100 mM NaF, 0.25% NP40, 1/100 PMSF, 1/100 phosphatase and protease inhibitors). Nuclear and cytosolic extracts were obtained as previously described.^47^ In brief, cells were lysed in buffer containing 10 mM HEPES, pH 7.9, 10 mM KCl, 1.5 mM MgCl_2_, 0.1 mM EDTA, 1 mM DTT, 0.25% Igepal CA-630, 1 mM PMSF, protease inhibitor cocktail (P8340, Sigma-Aldrich), and phosphatase inhibitor cocktail 2 and 3 (P5726 and P0044, Sigma-Aldrich). After centrifugation (10 min at 600 × *g*), the supernatant was saved as the cytoplasm extract, and the nuclear pellet was then suspended on ice in nuclear extract buffer containing 20 mM HEPES, pH 7.9, 420 mM NaCl, 1.5 mM MgCl_2_, 0.1 mM EDTA, 1 mM DTT, 25% glycerol, 1 mM PMSF, P8340, and P2850. Samples were then incubated for 15 min at 4 °C and centrifuged at 10 000 × *g* for 10 min and the supernatant was saved as the nuclear extract.

### Western blot analysis

Proteins were loaded onto SDS-PAGE gels containing 15% (LC3, caspase-3) or 10% (p62, Pp70S6K or AIF) acrylamide. After migration, the proteins were blotted onto Protran nitrocellulose membranes (10600003, GE Healthcare, Velizy-Villacoublay, France). The membranes were blocked by incubation for 1 h with 5% non-fat dried milk or BSA/TBS Tween-20 (0.1%), and then incubated overnight at 4 °C with the primary antibodies: LC3 (1/1000, NB100-2220, MBL International Corporation), p62 (1/1000, sc-28359, Santa Cruz Biotechnology), caspase-3 (1/1000, 9662, Cell Signaling, Danvers, MA, USA), Pp70SK6 (1/1000, 9205 S, Cell Signaling), p70S6K (1/1000, 9202 S, Cell Signaling), 4E-BP1 (1/1000, 9452, Cell Signaling), P4E-BP1 (1/1000, 2855, Cell Signaling), succinate dehydrogenase subunit A (SDHA) (1/1000, 5839, Cell Signaling), AIF (1/1000, sc-55519, Santa Cruz Biotechnology), and Histone H3 (1/1000, (1/1000, sc 56616, Santa Cruz Biotechnology). Membranes were then incubated with the corresponding secondary antibody, either goat anti-rabbit IgG-HRP (1/5000, sc-2030, Santa Cruz Biotechnology) or goat anti-mouse IgG-HRP (1/5000, sc-2031, Santa Cruz Biotechnology), for 1 h at room temperature. Secondary antibody binding was then detected by electrochemiluminescence. We checked for equal protein loading, by stripping the membranes and reprobing them with *β*-actin antibody (1/5000, A-5441, Sigma-Aldrich). The intensity of the immunoreactive bands was quantified by densitometry with Quantity one software from Bio-Rad (Hercules, CA, USA), and the results were expressed as a ratio with respect to *β*-actin immunoreactivity intensity.

### RNA extraction and reverse transcription-quantitative PCR

Total RNA was extracted from endothelial cells with the Nucleopsin RNA/protein kit (740933, Macherey-Nagel, Hoerdt, France), according to the manufacturer's recommendations. We generated cDNA by the reverse transcription of 0.5 *μ*g of total RNA in a final volume of 20 *μ*l with the A3500 reverse transcription system (Promega, Charbonnieres Les Bains, France). Quantitative PCR was then performed in duplicate with the CFX96 real-time PCR detection system (Bio-Rad) and the IQ SYBR Green supermix (170-8882, Bio-Rad), according to the manufacturer's protocol. The amplification of specific transcripts was confirmed by melting curve profiles generated at the end of the PCR program. The primers were designed with Primer Express software from Life Technologies (Supplementary Table 3). The values for each target gene were normalized with respect to the Ct value for rRNA *β*2M (ΔCt=Ct target gene – *Ctβ2M*). The relative amount of target mRNA is given by 2^−ΔΔCt^, where ΔΔCt=(ΔCt of target)−(ΔCt of control). Relative gene expression was normalized with respect to control levels, arbitrarily set to 1.0.

### Viability assay

Cell viability was assessed by calcein/ethidium labeling (L-3224, Life Technologies). Calcein is retained in living cells, which display esterase activity converting the almost nonfluorescent cell-permanent calcein AM into the highly fluorescent calcein. Ethidium homodimer-1 can enter cells and bind nucleic acids when the membranes are damaged. After treatment, the cells were washed three times with PBS and incubated for 45 min with calcein AM (1 *μ*M) and ethidium D1 (2 *μ*M) in PBS at room temperature. They were then observed under a Leica DMI-600B fluorescence microscope (Leica Microsystems), excitation/emission: 530/580 nm. In each experiment, we obtained six images for each set of conditions, from similar locations, and the results for these images were averaged for statistical analysis. Living cells were identified as displaying green fluorescence due to calcein AM only. Any cell displaying red fluorescence due to EthD-1 staining was considered to be dead or dying. The number of ethidium-labeled cells was determined from photomicrographs by image segmentation with ImageJ analysis software (Bethesda, MD, USA).

### Real-time videomicroscopy

Pups were killed by decapitation, and their brains were rapidly dissected for isolation of the cerebral hemispheres. The meninges covering the brains were carefully removed and the brains were immediately placed in aCSF. Transverse slices (250 *μ*m) were cut at 4 °C with a VT1000S vibratome (Leica Microsystems, Wetzlar, Germany). They were transferred to 24-well Costar plates containing aCSF and incubated for 30 min at 37 °C, in a humidified incubator, under an atmosphere containing 5% CO_2_ and 95% air. The slices were washed with fresh aCSF and incubated for 6 h at a constant temperature (37 °C), under a controlled atmosphere containing 5% CO_2_, on an inverted microscope (Leica microsystems) equipped with an incubation chamber (PeCon GmbH, Erbach, Germany), computer-controlled illumination shutters and filter wheels (Roper Scientific, Lisses, France). Slices were continuously perfused with aCSF with or without 50 mM ethanol and 200 nM rapamycin. Images were acquired with a × 10 objective at 30 min intervals over a 6 h period. Microvessel length was then determined from the acquired images with MetaMorph software (Sunnyvale, CA, USA).

### Mitochondrial membrane potential analysis

JC-1 stain from (Life Technologies) was used for labeling brain MPMVEC at 2 *μ*M concentration followed by incubation for 20 min at 37 °C. Labeled cells were imaged using a Leica DMI-600B fluorescence microscope (Leica Microsystems). Each group of cells (cells incubated with control, ethanol or acetaldehyde medium) were imaged using 488 nm excitation wavelength, and the emission signals were obtained through 525 and 590 emission channels at the same parameters of pinhole aperture and detector voltage.

### Statistical analyses

Data obtained from human brains were expressed as median interquartile range, and range of values and Mann–Whitney test was used to compare the number of immune-positive dots per cell and between pFAS/FAS and control groups. Data obtained *in vitro* and *ex vivo* were expressed as mean±S.E.M. The overall significance of the results obtained was examined using Student's paired *t*-test. Statistically significant differences between multiple comparisons were determined by a two-way analysis of variance with the appropriate Bonferroni correction for multiple comparisons test. Significance was defined by *P*-values <0.05. The statistical analyses were carried out with the biostatistical Prism software (GraphPad Software, La Jolla, CA, USA).
