# Supplementary material for: Prenatal alcohol exposure impairs autophagy in neonatal brain cortical microvessels
Source: Cell Death Dis. 2017 Feb 9;8(2):e2610–. doi: 10.1038/cddis.2017.29 (PMC5386476; doi:10.1038/cddis.2017.29)
Supplement: Supplementary Table 1 [file cddis201729x1.docx]

**Supplementary Table 1. Main clinical and morphological characteristics of control groups for brain studies.**

| **Control**  **(WG)** | **Cerebral maturation**  **(WG)** | **Cause of death** | **Medical termination** |
| --- | --- | --- | --- |
|  |  |  | **of pregnancy** |
| 29 | 22 | *Abruptio placentae*  Premature rupture of the membranes | No |
| 30 | 30 | Neonatal septicemia (Escherichia Coli) | No |
| 31 | 31 | Hypovolemic collapse | No |
| 34 | 34 | Autosomal recessive polycystic kidney disease | No |

WG: weeks' gestation
