# Supplementary material for: Prenatal alcohol exposure impairs autophagy in neonatal brain cortical microvessels
Source: Cell Death Dis. 2017 Feb 9;8(2):e2610–. doi: 10.1038/cddis.2017.29 (PMC5386476; doi:10.1038/cddis.2017.29)
Supplement: Supplementary Table 2 [file cddis201729x2.docx]

**Supplementary Table 2. Main clinical and morphological characteristics of pFAS/FAS group of patients for brain studies.**

| **pFAS/FAS**  **WG** | **Cause of death** | **IUGR** | **Maternal alcohol intake** | **Maternal**  **Co-morbidity** |
| --- | --- | --- | --- | --- |
| 29 | IUFD  Polymalformative syndrome | 5^th^ percentile  IUGR | Daily chronic alcohol intake* | Increased MGV and γGT |
| 30 | IUFD  *Abruptio placentae* | 10^th^ percentile | Daily chronic alcohol intake** | Cannabis addiction  Treated hypothyroidism |
| 31 | IUFD  Preeclampsia | 3^rd^ percentile  IUGR | Daily chronic alcohol intake* | Increased MGV and γGT |
| 33 | IUFD  Acute alcohol intoxication | 50^th^ percentile | Chronic and Binge drinking*  (4.98 g/L) | Multi-drug addiction  Increased MGV and γGT  First pregnancy: IUFD at 33 WG  One child alive with FAS |

WG: weeks' gestation; IUFD: *in utero* fetal death; IUGR: intra uterine growth retardation; MGV: mean globular volume; γGT: gamma-glutamyl transferase; * maternal self-report; ** suspected.
