# Supplementary material for: Prenatal alcohol exposure impairs autophagy in neonatal brain cortical microvessels
Source: Cell Death Dis. 2017 Feb 9;8(2):e2610–. doi: 10.1038/cddis.2017.29 (PMC5386476; doi:10.1038/cddis.2017.29)
Supplement: Supplementary Table 3 [file cddis201729x3.docx]

**Supplemental Table 3: Forward and reverse PCR primers based on the sequence of the mouse gene**

| **Gene** | **Sequence 5' to 3'** |
| --- | --- |
| *LC3* F | TTTCTGGTCCCAGACCATGT |
| *LC3* R | GTTGACCAGCAGGAAGAAGG |
| *Beclin* F | CAGGCGAAACCAGGAGAG |
| *Beclin* R | CGAGTTTCAATAAATGGCTCCT |
| *Atg5* F | TCAACCAAAGCCAAACCGAG |
| *Atg5* R | GTGATCCCGGCAGACAGAAC |
| *Atg3* F | GAGGCTACCCTAGACACAAGG |
| *Atg3* R | GGCTGCCGTTGCTCATCATA |
| *Atg7* F | CCGGTGGCTTCCTACTGTTA |
| *Atg7* R | AAGGCAGCGTTGATGACC |
| *p62* F | AGGATGGGGACTTGGTTGC |
| *p62* R | TCACAGATCACATTGGGGTGC |
| *B2M* F | GCCGAACATACTGAACTGCTAC |
| *B2M* R | GCTGAAGGACATATCTGACATCTC |
